#  Identifying the associated risks of pneumonia in COPD patients: ARCTIC an observational study

**DOI:** 10.1186/s12931-018-0868-y

**Published:** 2018-09-10

**Authors:** Christer Janson, Gunnar Johansson, Björn Ställberg, Karin Lisspers, Petter Olsson, Dorothy L. Keininger, Milica Uhde, Florian S. Gutzwiller, Leif Jörgensen, Kjell Larsson

**Affiliations:** 1 Department of Medical Sciences: Respiratory, Allergy and Sleep Research, Uppsala University, Akademiska sjukhuset, 75185 Uppsala, Sweden; 2 Department of Public Health and Caring Sciences, Family Medicine and Preventive Medicine, Uppsala University, Uppsala, Sweden; 3 Novartis Sverige AB, Täby, Sweden; 4 Novartis, Basel, Switzerland; 5 IQVIA, Stockholm, Sweden; 6 IQVIA, Copenhagen, Denmark; 7 Karolinska Institutet, Solna, Sweden

**Keywords:** Chronic obstructive pulmonary disease, Inhaled corticosteroids, Pneumonia, Sweden, Comorbidities, Asthma

## Abstract

**Background:**

Inhaled corticosteroids (ICS) are associated with an increased risk of pneumonia in patients with chronic obstructive pulmonary disease (COPD). Other factors such as severity of airflow limitation and concurrent asthma may further raise the possibility of developing pneumonia. This study assessed the risk of pneumonia associated with ICS in patients with COPD.

**Methods:**

Electronic Medical Record data linked to National Health Registries were collected from COPD patients and matched reference controls in 52 Swedish primary care centers (2000–2014). Levels of ICS treatment (high, low, no ICS) and associated comorbidities were assessed. Patients were categorized by airflow limitation severity.

**Results:**

A total of 6623 patients with COPD and 48,566 controls were analyzed. Patients with COPD had a more than 4-fold increase in pneumonia versus reference controls (hazard ratio [HR] 4.76, 95% confidence interval [CI]: 4.48–5.06). ICS use increased the risk of pneumonia by 20–30% in patients with COPD with forced expiratory volume in 1 s ≥ 50% versus patients not using ICS. Asthma was an independent risk factor for pneumonia in the COPD population. Multivariate analysis identified independent predictors of pneumonia in the overall population. The highest risk of pneumonia was associated with high dose ICS (HR 1.41, 95% CI: 1.23–1.62).

**Conclusions:**

Patients with COPD have a greater risk of pneumonia versus reference controls; ICS use and concurrent asthma increased the risk of pneumonia further.

## Background

International recommendations for the treatment of patients with chronic obstructive pulmonary disease (COPD) restrict the use of inhaled corticosteroids (ICS) containing treatments in patients at high risk of exacerbation (Global initiative for chronic Obstructive Lung Disease [GOLD] Groups C and D) or patients with asthma-COPD overlap (ACO) [1]. Although ICS-containing treatments are not recommended for patients at low risk of exacerbation (GOLD Groups A and B), they are widely prescribed [2–4]. Until 2015, Swedish national guidelines only recommended the use of ICS in combination with long-acting beta-2 agonists (LABA) for patients with COPD with a forced expiratory volume in 1 s (FEV_1_) < 50% predicted who were experiencing exacerbations [5]. However, real-world studies indicate that ICS/LABA are often used in Swedish patients with a FEV_1_ ≥ 50% [6].

Associations between ICS use and several adverse effects in patients with COPD were first observed in the TORCH (Towards a Revolution in COPD Health) trial, in particular an increased risk of pneumonia [7], which was most apparent in patients with mild-to-moderate airflow limitation. Further trials demonstrated an association between ICS use and an increased risk of pneumonia [8–12]. Meta-analyses have also confirmed an increase in the risk of pneumonia with ICS use; however, no increased risk of mortality from pneumonia was observed [13, 14]. An association between use of ICS and pneumonia has also been found in observational studies using data from electronic medical records (EMRs) and registries, but these studies have largely lacked spirometry data [15, 16].

Asthma is a common comorbidity in COPD and the term “ACO”, (patients who have characteristics of both asthma and COPD), has been presented in international recommendations [1, 17]. Notably, several studies found that patients with ACO have more exacerbations and a lower health-related quality of life than patients with COPD alone [18, 19], suggesting that patients who suffer from ACO, are a particularly vulnerable group. To our knowledge, no studies have compared the risk of pneumonia in patients with ACO with COPD only patients.

We analyzed data from a real-world study of Swedish primary care patients with COPD (the ARCTIC observational study) to identify risk factors for pneumonia and determine any relationship with ICS use. Additional aims assessed how airflow limitation severity (measured by FEV_1_) and the presence of concurrent asthma affected the risk of pneumonia in patients with COPD. We anticipated that the risk of pneumonia would be higher among patients with COPD taking ICS compared with COPD patients not taking ICS and the reference controls.

## Methods

### Study design

ARCTIC was a retrospective, observational cohort study of longitudinal patient-level data extracted from the EMRs of Swedish primary care patients. The objectives of the ARCTIC study were to generate evidence to better manage patients with COPD, to foster early diagnosis, and to characterize treatment patterns and associated outcomes. This study was conducted in accordance with the principles of the Declaration of Helsinki and ethical approval was granted by the ethics review board at Uppsala University, Sweden (number: 2014–397).

The general population in Sweden is approximately 10 million [20]. Data were collected from patients with physician-diagnosed COPD and reference patients in 52 primary care centers covering approximately 200,000 patients between the years 2000–2014 using an established software system (Pygargus Customized eXraction Program, CXP 3.0), and included: age, gender, prescriptions (according to the World Health Organization Anatomic Therapeutic Chemical [ATC] codes), disease diagnoses (according to the International Classification of Disease codes [ICD-10 codes]), spirometry measurements (FEV_1_ values), laboratory tests, healthcare professional (HCP) visits, and referrals. EMR data were linked by the Swedish National Board of Health and Welfare using individual patient identification (ID) numbers to National Registry data sources (patient IDs were pseudonymized): (i) the Longitudinal Integration Database for Health Insurance and Labour Market Studies (LISA [21]), which includes socio-demographic data including educational level, marital status and family situation, occupational status, retirement, economic compensation and social benefits; (ii) the National Patient Register [22], which contains data relating to diagnosis (ICD-10 code and associated position), surgery, gender, age, region, hospital visits, specialty visits, hospital admissions and discharges, and medical procedures and surgeries performed in the inpatient and outpatient specialist settings; (iii) the National Prescription register [22], which tracks full details of all dispensed medications (ATC codes), including brand name, prescription date, dose, strength, pack size, specialty of the prescriber and costs associated with the drug prescription; and (iv) the Cause of Death Register [22], which holds information on social security number, home district, sex, date of death and cause of death.

### Study subjects

COPD is rare under the age of 40, therefore, patients eligible for inclusion were those aged ≥40 years with lung function measurements and who had received a doctor’s diagnosis of COPD (ICD-10 code: J44), and/or asthma (ICD-10 code: J45/J46) in the primary care setting (EMR database) that was then verified as COPD only or COPD and asthma in a hospital setting (according to the National Patient Register). Patients’ diagnoses were defined by ICD codes, while lung function was used to assess the degree of airflow limitation based on data collected from EMRs and the National Patient Register. The first patient to receive a COPD diagnosis was in 2000 (index date). An age- and gender-matched reference population was selected from the primary care centers, excluding those who had a diagnosis of COPD and/or asthma. The matching criteria for patients and the reference group included, age, gender and the starting year for the index date. The index date for the reference group was selected as a random date between the start and end of the observation period for the reference patients. To focus exclusively on the effects of ICS, subjects who had taken two or more prescriptions of oral corticosteroids from 2005 to 2014 were excluded. Each COPD patient was matched with a mean of seven reference patients, depending on the size of the age group, to allow for comparisons in the associated risks for pneumonia, with emphasis on ICS use. Patients in the control group were not allowed to take ICS. ICS use was established using the ATC code R03BA from the EMRs and patient registries. The COPD and reference group patients were stratified by the level of ICS exposure after the index date (high dose: ≥800 μg/day budesonide or equivalent; low dose: < 640 μg/day budesonide or equivalent).

### Outcomes

The main outcome of interest was time-to-first pneumonia diagnosis, identified using ICD codes J12–J20. Patients were also categorized by airflow limitation severity: 1) no spirometry data available; 2) FEV_1_ < 50% predicted; and 3) FEV_1_ ≥ 50% predicted. These categories were used to stratify results.

### Statistical analysis

Data were analyzed concerning wrong personal identifiers and wrong dates, and outliers’ analyses were conducted for numeric variables. There was no imputation of missing data; these were reported in the descriptive analysis (the exception being when the day of the month was missing in order to keep the data anonymous, the day of the month was assumed to be the 15th). Patient demographics were described for both patients with COPD and reference controls. Time-to-first pneumonia event analyses were conducted using Cox regression models with 95% confidence intervals (CI) in order to determine the risk of pneumonia associated with varying levels of ICS use, disease states and airflow limitation severity. The statistical reference group for analysis was patients without COPD or asthma who were not taking ICS, except when analyzing the use of ICS, where the reference group included COPD patients not using ICS. The analyses were stratified based on the disease status (asthma with no COPD, asthma and COPD, and COPD with no asthma), level of ICS use (high, low, and no ICS) and the level of airflow limitation severity (no spirometry data available, FEV_1_ < 50% predicted, and FEV_1_ ≥ 50% predicted). Finally, risk factors for pneumonia in the COPD group were analyzed in a multivariate model, which included the variables that were statistically associated with pneumonia in the stratified analyses (*p* < 0.05). No immortal time bias was identified in our data. All analyses were performed using SAS version 9.3 or newer (SAS Institute Inc., Cary, NC) statistics software.

A sample size calculation was conducted for the ARCTIC trial before the start of the study, similar to that of another observational matched cohort study in patients with COPD [15]. The power calculation ensured that a sufficiently large sample was obtained to address the study’s primary research questions, while also ensuring a large enough sample to address the additional planned sub-analyses. Given the large number of research questions and outcomes of interest, the power calculation was not based on a specific outcome. To achieve a power of 80% to detect a 4% between-group difference at a 5% significance level, 13,800 patients were required. A target sample size of 15,000 patients with COPD was therefore established before the start of the study.

## Results

### Patient demographics

**Fig. 1:**
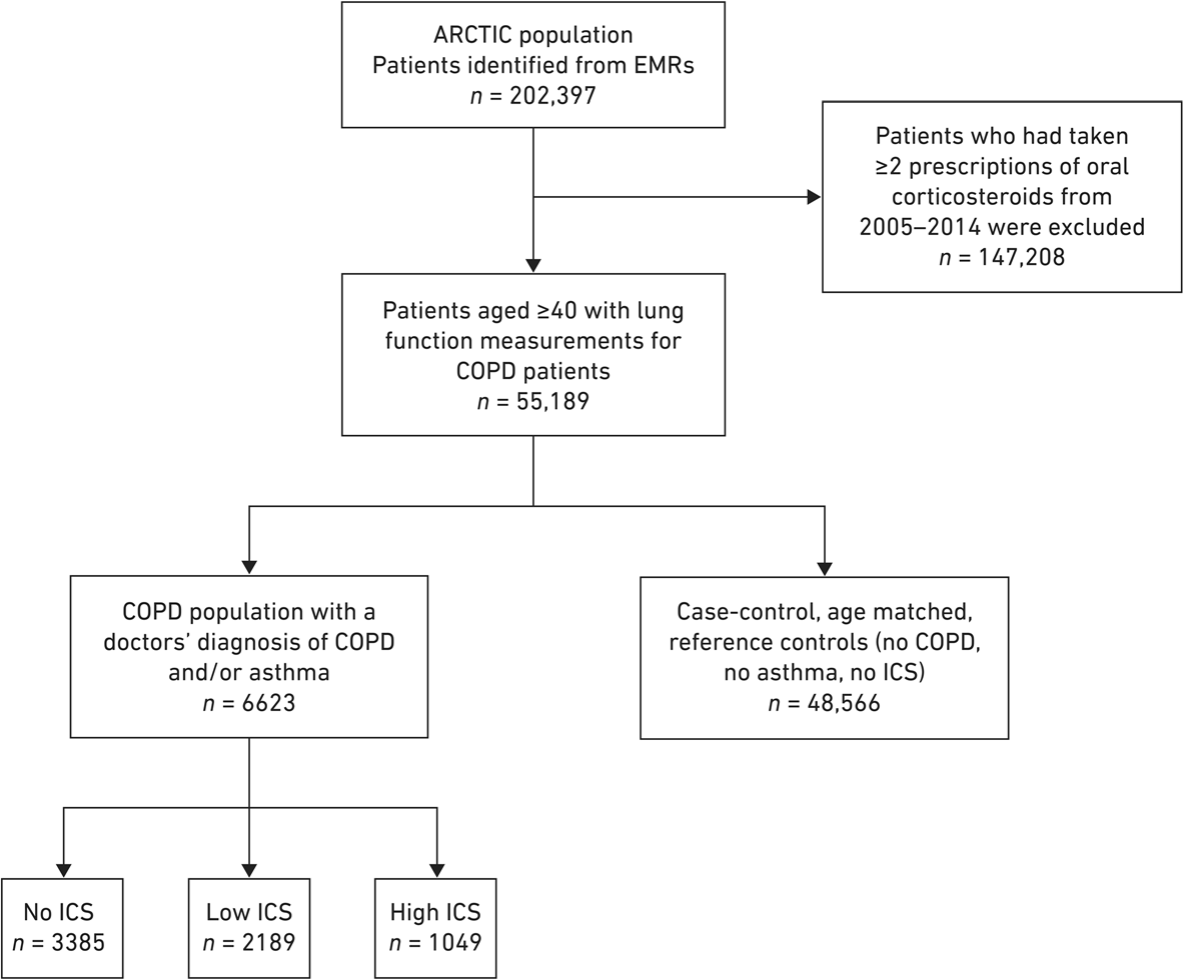
Study cohorts and criteria for patients with a doctor’s diagnosis of COPD and/or asthma. *COPD* chronic obstructive pulmonary disease; *EMRs* electronic medical records; *ICS* inhaled corticosteroids (low dose ICS: < 640 μg/day; high dose ICS: ≥800 μg/day)

From a total of 55,189 patients listed in EMRs with lung function measurements for patients with COPD, 6623 patients with COPD and/or asthma were identified as eligible for inclusion in this study, matched with 48,566 reference controls (Fig. 1). Baseline characteristics for both study populations are presented in Table 1. While the populations were well matched for gender, patients in the COPD population were older than those in the reference population (66 vs. 65 years, *p* < 0.0001) and had significantly higher levels of healthcare utilization, comorbidities and rescue medication use in comparison with the reference controls, all of which were adjusted for in the comparative analyses (Table 1). For example, 38.0% of patients with COPD had cardiovascular disease compared with 20.4% of the reference controls (*p* < 0.0001). The mean FEV_1_ in the COPD population was 58.6 ± 20.1% predicted.

**Table 1:** Baseline patient demographics for patients with lung function measurements and reference controls^a^ without ICS usage

Variable	COPD with lung function data (*N* = 6623)	Reference, without ICS usage (*N* = 48,566)	*p*-value
Age, mean years ± SD	65.9 ± 10.1	64.5 ± 10.5	< 0.0001
Female, *n* (%)	3688 (55.7)	26,792 (55.2)	0.4699
Comorbidities below, n (%)			
Asthma, J45	974 (14.7)	0	< 0.0001
Cardiovascular disease, I00-I99	2514 (38.0)	9932 (20.4)	< 0.0001
Hypertensive diseases, I10-I15	1707 (25.8)	5941 (12.2)	< 0.0001
Ischemic heart diseases, I20-I25	584 (8.8)	2052 (4.2)	< 0.0001
Cerebrovascular diseases, I60-I69	213 (3.2)	1182 (2.4)	0.0001
Diabetes Type I, E10	83 (1.2)	757 (1.6)	0.0568
Diabetes Type II, E11 + E13	418 (6.3)	2049 (4.2)	< 0.0001
Hyperlipidemia, E78.5	161 (2.4)	502 (1.0)	< 0.0001
Depression, F32 + F33	456 (6.9)	873 (1.8)	< 0.0001
Osteoporosis, M80 + M81	139 (2.1)	402 (0.8)	< 0.0001
Fractures, S2	356 (5.4)	1968 (4.0)	< 0.0001
Charlson Comorbidity Index value, mean ± SD	1.55 ± 0.8	1.26 ± 0.6	< 0.0001
Health care utilization			
Number of outpatient hospital visits/year in 2 years before index date, mean ± SD	1.53 ± 2.4	1.60 ± 3.7	0.1980
Number of contacts to primary care/year in 2 years before index date, mean ± SD	12.0 ± 16.0	4.14 ± 13.6	< 0.0001
ICS use, n (%)			
No ICS	3385 (51.1)	NA	
Low dose ICS^b^	2189 (33.0)	NA	
High dose ICS^c^	1049 (15.8)	NA	

^a^Patients in the reference control group were excluded if they had a diagnosis of COPD and/or asthma and did not take ICS; ^b^Low dose ICS: < 640 μg/day; ^c^High dose ICS: ≥800 μg/day

*COPD* chronic obstructive pulmonary disease, *ICS* inhaled corticosteroids, *NA* not applicable

### Medication use

Thirty-three percent of patients with COPD were using low dose ICS, while 16% were receiving high dose ICS (Table 1). The majority of patients taking ICS used budesonide (71.5%), whereas only 7.3% used fluticasone propionate (Table 2). Of those patients included in the study, 71.7% had not used oral corticosteroids and 28.3% had collected one prescription.

**Table 2:** Types of inhaled corticosteroids used by the COPD population. Reference patients did not use ICS

Variable	COPD patients with lung function data (*N* = 6623)^a^
Types of ICS, *n* (%)	
Budesonide	2317 (71.5)
Fluticasone propionate	236 (7.3)
Budesonide/fluticasone propionate^b^	655 (20.2)
Other	30 (0.9)

^a^No ICS *n* = 3385; ^b^Includes patients that switched from budesonide to fluticasone propionate and fluticasone propionate to budesonide during the study period, as well as a few patients that were using both budesonide and fluticasone propionate at the same time

*COPD* chronic obstructive pulmonary disease, *ICS* inhaled corticosteroids

### Pneumonia risk

The diagnosis of pneumonia was collected from primary and secondary care settings. During the follow up (2000–2014), 2324 (35.1%) of COPD patients had at least one episode of pneumonia compared with 5036 (10.4%) in the reference population (*p* < 0.0001). Overall, patients with COPD had a more than 4-fold increase in risk of pneumonia than the reference controls (hazard ratio [HR] 4.76, 95% CI: 4.48–5.06). The risk of pneumonia was higher in men compared with women, both in those with more severe and less severe airflow obstruction (FEV_1_ < 50%: HR 1.28, 95% CI: 1.20–1.36; FEV_1_ ≥ 50%: HR 1.26, 95% CI: 1.19–1.34). Furthermore, for every 1 year increase in age there was a 4% increase in risk of pneumonia (Table 3).

**Table 3:** Hazard ratio for pneumonia in COPD patients stratified by FEV_1_

	FEV_1_ < 50% Hazard ratio (95% CI) (*N* = 2730)	FEV_1_ ≥ 50% Hazard ratio (95% CI) (*N* = 5547)
Age^a^	1.04 (1.03–1.04)	1.04 (1.04–1.04)
Males^b^	1.28 (1.20–1.36)	1.26 (1.19–1.34)
COPD + asthma compared with reference^c^		
Reference	1	1
No ICS	3.06 (2.35–3.97)	4.61 (3.70–5.75)
Low ICS^d^	6.61 (5.43–8.05)	5.31 (4.57–6.18)
High ICS^e^	6.40 (5.30–7.72)	5.40 (4.56–6.38)
‘COPD without asthma’ compared with reference^c^		
Reference	1	1
No ICS	4.35 (3.79–4.99)	4.01 (3.58–4.49)
Low ICS	6.15 (5.23–7.24)	4.52 (3.91–5.23)
High ICS	4.91 (3.82–6.31)	4.62 (3.45–6.18)
‘COPD without asthma’: ICS use compared with no ICS use in the COPD population		
No ICS	1	1
Low ICS	1.06 (0.91–1.25)	1.20 (1.05–1.38)
High ICS	0.98 (0.81–1.17)	1.31 (1.10–1.56)

^a^Increased risk for every one year increase in age; ^b^Increased risk for males compared to females; ^c^Reference population (*n* = 48,566); case matched population with no asthma or COPD but with lung function measurements; ^d^Low dose ICS: < 640 μg/day; ^e^High dose ICS: ≥800 μg/day

*CI* confidence intervals, *COPD* chronic obstructive pulmonary disease, *FEV*_*1*_ forced expiratory volume in 1 s, *ICS* inhaled corticosteroids

### ICS use

**Fig. 2:**
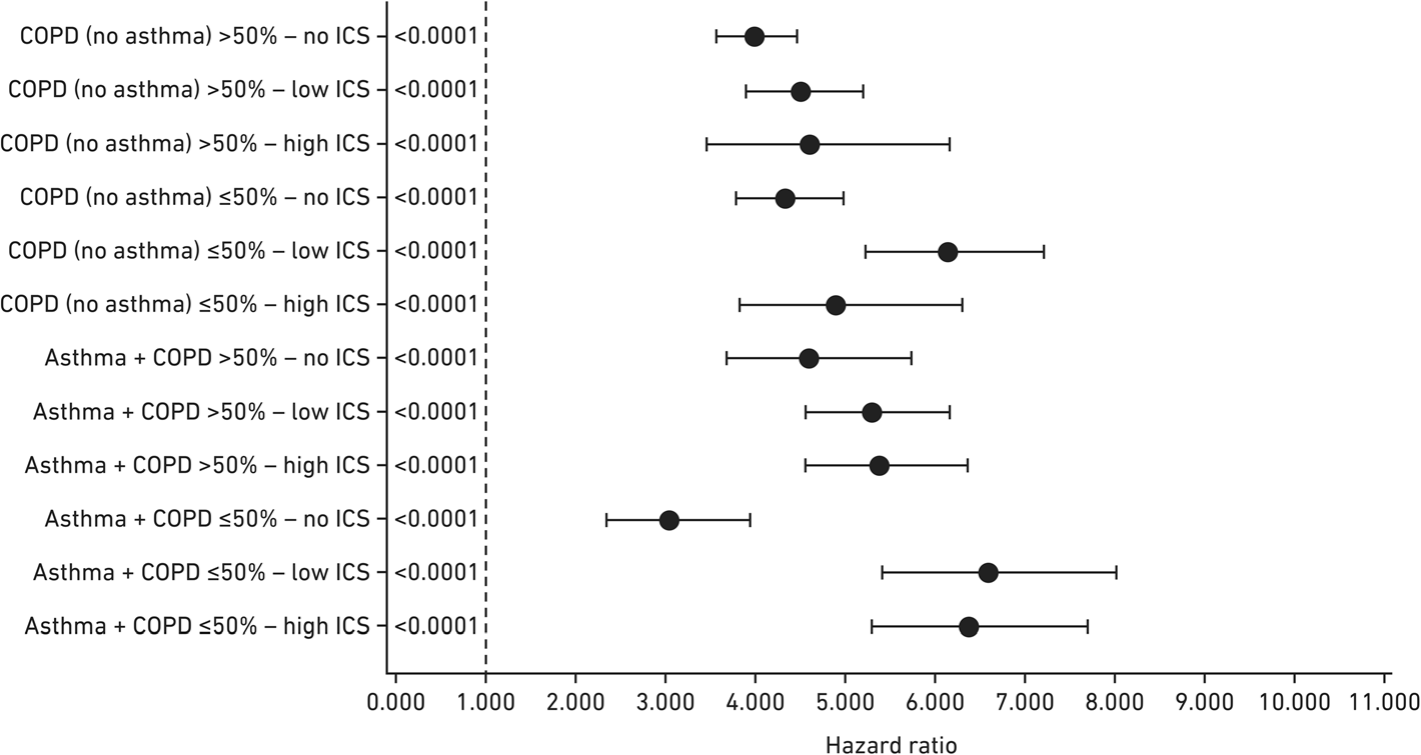
Forest plot showing the HR for pneumonia in COPD and/or asthma patients versus reference population*. *(No COPD and/or asthma, no ICS). All results were statistically significant, *p* < 0.0001. HR above 1 is an increased risk of pneumonia. *COPD* chronic obstructive pulmonary disease; *HR* hazard ratio; *ICS* inhaled corticosteroids (low dose ICS: < 640 μg/day; high dose ICS: ≥800 μg/day)

COPD was associated with an increased risk of pneumonia irrespective of ICS use (Table 3, Fig. 2). However, ICS use further increased the risk of pneumonia 5-fold among patients with COPD and asthma and in patients with FEV_1_ < 50% or ≥ 50% when the results were stratified by lung function (Table 3). ICS use was associated with a 20–30% increased risk of pneumonia in patients with COPD with FEV_1_ ≥ 50% compared with patients who were not using ICS.

### Presence of asthma

Overall, patients with concurrent asthma had a higher risk of pneumonia compared with those without asthma (Tables 3 and 4). However, the pattern of risk by ICS use is similar in patients with concurrent asthma and those with COPD alone. For example, in both the low and high dose ICS groups, ICS use was associated with an increased risk of pneumonia (Table 4).

**Table 4:** Hazard ratio for pneumonia in COPD patients stratified by presence of asthma

	COPD without asthma Hazard ratio (95% CI) (*N* = 4299)	COPD with asthma Hazard ratio (95% CI) (*N* = 2324)
Age^a^	1.05 (1.05–1.06)	1.05 (1.05–1.05)
Males^b^	1.19 (1.12–1.27)	1.21 (1.14–1.29)
Reference population^c^	1	1
COPD with no ICS versus ref	3.35 (2.82–3.97)	5.00 (4.56–5.48)
COPD with low ICS^d^ versus ref	7.86 (6.96–8.89)	6.36 (5.69–7.11)
COPD with high ICS^e^ versus ref	7.08 (6.22–8.04)	4.56 (3.76–5.53)
Within the COPD group: ICS use compared with no ICS use		
No ICS	1	1
Low ICS	1.29 (1.05–1.58)	1.46 (1.28–1.67)
High ICS	1.59 (1.30–1.96)	1.69 (1.37–2.07)

^a^Increased risk for every 1 year increase in age; ^b^Increased risk for males compared to females; ^c^Reference population = case matched population with no asthma or COPD but with lung function measurements; ^d^Low dose ICS: < 640 μg/day; ^e^High dose ICS: ≥800 μg/day

*CI* confidence intervals, *COPD* chronic obstructive pulmonary disease, *ICS* inhaled corticosteroids, *ref* reference

### Independent variables associated with pneumonia

A multivariate analysis was carried out to identify independent predictors of pneumonia in patients with COPD. The analysis showed that variables including FEV_1_, gender, the use of ICS and presence of asthma were significantly independently associated with an increased risk of a pneumonia event (Table 5). Further, a dose response for ICS was demonstrated, in which the highest risk of pneumonia was associated with the high dose of ICS (high ICS: HR 1.41, 95% CI: 1.23–1.62]; low ICS: HR 1.23, 95% CI: 1.10–1.38]). In addition, no significant association was found between the Charlson Comorbidity Index (CCI) and pneumonia. There was also no significant independent association found between diabetes type II and pneumonia when the CCI was replaced with diabetes type II (HR 0.95, 95% CI: 0.74–1.23).

**Table 5:** HR for pneumonia in COPD patients only, including FEV_1_ and comorbidities in a multivariate model

	Hazard ratio (95% CI)	*p*-value
Age^a^	1.01 (1.00–1.01)	0.06
Male^b^	1.13 (1.03–1.25)	0.01
No ICS	1	
Low ICS^c^	1.23 (1.10–1.38)	0.0003
High ICS^d^	1.41 (1.23–1.62)	< 0.0001
FEV_1_ < 50%	1.33 (1.21–1.47)	
FEV_1_ ≥ 50%	1	
No asthma	1	
Asthma	1.13 (1.01–1.27)	0.0310
Charlson Comorbidity index^e^	1.02 (0.96–1.09)	0.4621

^a^Increased risk for every 1 year increase in age; ^b^Increased risk for males compared to females; ^c^Low dose ICS: < 640 μg/day; ^d^High dose ICS: ≥800 μg/day; ^e^For each one unit increase in Charlson Comorbidity index

*CI* confidence intervals, *COPD* chronic obstructive pulmonary disease, *FEV*_*1*_ forced expiratory volume in 1 s, *HR* hazard ratio, *ICS* inhaled corticosteroids

## Discussion

This retrospective real-world study in over 6000 primary care patients with a diagnosis of COPD and/or asthma aged ≥40 years has demonstrated that COPD increases the risk of pneumonia, and that the use of ICS further increases this risk. The presence of concurrent asthma may also be an influential risk factor for pneumonia.

### Pneumonia risk and the use of ICS

A higher risk of pneumonia while using ICS has been observed in other randomized clinical trials including the TORCH and Investigating New Standards for Prophylaxis in Reducing Exacerbations (INSPIRE) studies [7, 10, 23, 24]. However, some studies have also demonstrated the opposite effect [25, 26]. In this study, the risk of pneumonia associated with ICS use was lower in comparison with previous reports [16]. This could be related to the low proportion of patients using fluticasone propionate, since previous data suggests its use is associated with a particular increase in the risk of pneumonia. For example, a population-based cohort study showed that patients receiving fluticasone had a higher incidence rate and a higher risk of pneumonia than patients receiving budesonide (12.11 per 100 person-years vs. 10.65 per 100 person-years, adjusted HR 1.13, 95% CI: 1.08–1.20) [27]. An observational study also showed that the rate of serious pneumonia was doubled with fluticasone propionate (rate ratio [RR] 2.01; 95% CI: 1.93–2.10) and increased with the daily dose. In contrast, budesonide was associated with a 17% increase in rate, with no evidence of a dose response effect [15, 16]. However, evidence for the intra-class differences between ICS compounds in pneumonia risk has been disputed due to the lack of prospective randomized head to head studies [28].

### Severity of airflow limitation

Severity of airflow limitation may be an important factor in pneumonia risk, in that patients receiving ICS with severe-to-very severe airflow limitation (i.e. FEV_1_ < 50% predicted), had a higher risk of pneumonia than those with FEV_1_ ≥ 50%, regardless of ICS dose. This is in accordance with other studies [29]. However, in the ‘COPD without asthma’ patient group, the association between ICS use and pneumonia was stronger in those with an FEV_1_ ≥ 50% predicted (low ICS: HR 1.20; high ICS: HR 1.31, both vs. no ICS use) than in patients with an FEV_1_ < 50% (low ICS: HR 1.06; high ICS HR 0.98, both vs. no ICS use). This result was unexpected and of concern, since these patients were being treated outside the recommendations in Sweden. This finding is not in line with data from the TORCH study [29] and the reason for this is unclear. It could be argued that, although those with severely impaired airflow limitation appear to be at an increased risk of pneumonia, the impact of ICS on the risk in these patients is overestimated, and that patients who require ICS therapy with severely impaired lung function are already predisposed to respiratory tract infections. The results could also represent a survivor effect, in which patients with an FEV_1_ < 50% who may have been at increased risk of pneumonia at higher doses of ICS are no longer alive.

### Presence of asthma

In this study, the presence of asthma was an independent risk factor for pneumonia in the COPD population, and was associated with a 13% increase in the risk of pneumonia (HR 1.13, 95% CI: 1.01–1.27). This is consistent with results from previous studies. For example, in a case-control study in four Dutch healthcare centers, a history of asthma was independently associated with an increased risk of community-acquired pneumonia [30]. The study found that asthma was the strongest independent risk factor for pneumonia in both children (odds ratio [OR] 3.57, 95% CI: 1.86–6.88) and young adults (OR 2.69, 95% CI: 1.23–5.88) [30]. To our knowledge, no previous data on the risk of pneumonia in patients with asthma and COPD are available. However, other studies have identified that patients with COPD and concomitant asthma have more exacerbations and more severe airflow limitation compared with patients with COPD alone [18]. This indicates that patients with both COPD and co-existing asthma are a vulnerable patient group who may require distinct clinical management and surveillance.

### Health care utilization and comorbidities

We also found that patients with COPD had significantly higher levels of healthcare utilization and comorbidities than reference controls. Thirty-eight percent of patients had cardiovascular disease and 26% had hypertension. These rates are similar to those reported in other studies [31, 32]. The prevalence of asthma in patients with COPD in this study was higher (15%) compared to the other comorbidities but was low in comparison to other COPD studies, where concomitant asthma was reported in up to 40% of all patients with COPD [33]. In our study, the higher rates of comorbidities in patients with COPD compared to the reference population highlight the importance of a thorough assessment of patients with COPD by physicians and of taking these co-existing conditions into consideration when managing these patients. This will help ensure that delays in COPD diagnosis due to misdiagnosis by HCPs are reduced and that the correct treatment is given. However, further research is needed to understand the full impact of different co-morbidities, including asthma, on COPD outcomes.

### Strengths and limitations

This study has a number of strengths. The large sample size of patients with COPD with spirometry data from a primary care setting and real-world study design provides a unique set of data that are reflective of the general patient population. The inclusion of a reference population, several disease states, severity of airflow limitation, concurrent asthma and ICS use (stratified by low, high or no ICS use) are all further strengths of this study, given that only one other cohort study has stratified ICS use [16].

A potential limitation to this study is the retrospective study design, which introduces the potential for bias and confounding due to variables that may not have been accounted for in our analysis. A further limitation was that no investigation into mortality was made. One study demonstrated that mortality rate was approximately 2–6 times higher in patients with COPD compared with the general Swedish population [34]; however, there was no increase in death in those using ICS [35]. There was also no information on smoking or body mass index (BMI). A previous multivariate analysis found that, regardless of treatment, a BMI of < 25 kg/m^2^ was a risk factor for pneumonia [24]. We also lacked patient reported outcome data such as the COPD Assessment Test (CAT) and could therefore not use the GOLD A, B, C, D grading system [36]. Furthermore, as the diagnosis of pneumonia was carried out in primary and secondary care, it could be argued that the diagnosis of pneumonia was only accurate in secondary care settings, where chest radiographs and X-rays are used in all patients with suspected pneumonia. Evidence from diagnostic studies also suggests that a diagnosis of pneumonia in general practice is associated with reduced accuracy [37]. Since only Swedish patients were enrolled in this study, the generalizability of results may be limited to patients in other parts of the world. In addition, variability in regional healthcare systems, criteria and practices between and within medical centers make it difficult to compare data [38]. However, we believe our findings have important clinical implications and demonstrate the association between ICS and pneumonia in patients with COPD. As a result, physicians should prescribe ICS judiciously in patients with COPD, particularly taking into account the presence of comorbidities such as asthma.

### Clinical implications

Evidence has shown that indacaterol/glycopyrronium, a fixed-dose LABA/long-acting muscarinic antagonist (LAMA) combination, was superior to the ICS/LABA salmeterol/fluticasone in preventing exacerbations and improving patient reported outcomes in patients with COPD with moderate-to-very severe airflow limitation with or without a history of exacerbations [39–41]. Thus, since ICS increases the risk of pneumonia, a LABA/LAMA combination may be an appropriate first choice treatment [39–41]. It is therefore important to understand which patients may benefit from ICS in order to reduce unnecessary exposure of patients to ICS-associated risks. Further studies are needed to determine who should be treated with ICS-containing regimens, focusing on both the benefits and risks, to improve understanding and aid physician decision making.

## Conclusion

Despite its limitations, this large-scale primary care study provides important insights into the characteristics of patients with COPD in a real-world setting. We have demonstrated that patients with COPD are at high risk of pneumonia and that the use of ICS and the presence of concomitant asthma are related to a further increase in the risk of pneumonia. Such insights should inform the management of COPD by primary care physicians in order to maximize the chances of positive outcomes among the patients they treat.

## Abbreviations

ACO: Asthma-COPD overlap
ATC: Anatomic Therapeutic Chemical
BMI: Body mass index
CI: Confidence intervals
COPD: Chronic obstructive pulmonary disease
EMRs: Electronic medical records
FEV_1_: Forced expiratory volume in 1 s
GOLD: Global initiative for chronic Obstructive Lung Disease
HCP: Healthcare professionals
HR: Hazard ratio
ICD: International Classification of Disease
ICS: inhaled corticosteroids
ID: Identification
INSPIRE: Investigating New Standards for Prophylaxis in Reducing Exacerbations
LABA: Long-acting beta-2 agonists
LAMA: Long-acting muscarinic antagonist
LISA: Longitudinal Integration Database for Health Insurance and Labour Market Studies
OR: Odds ratio
RR: Rate ratio
TORCH: Towards a Revolution in COPD Health
